# nc886, a Non-Coding RNA, Is a New Biomarker and Epigenetic Mediator of Cellular Senescence in Fibroblasts

**DOI:** 10.3390/ijms222413673

**Published:** 2021-12-20

**Authors:** Yuna Kim, Hyanggi Ji, Eunae Cho, Nok-Hyun Park, Kyeonghwan Hwang, Wonseok Park, Kwang-Soo Lee, Deokhoon Park, Eunsun Jung

**Affiliations:** 1 Biospectrum Life Science Institute, A-1805, U-TOWER, Yongin-si 16827, Korea; bioyn@biospectrum.com (Y.K.); biocr@biospectrum.com (H.J.); biozr@biospectrum.com (E.C.); bioyc@biospectrum.com (K.-S.L.); pdh@biospectrum.com (D.P.); 2 Basic Research and Innovation Division, Amorepacific Corporation R&D Center, Youngin-si 17074, Korea; aquareve@amorepacific.com (N.-H.P.); khhwang@amorepacific.com (K.H.); wspark@amorepacific.com (W.P.)

**Keywords:** fibroblasts, replicative senescence, epigenetic regulation, nc886, green tea extract

## Abstract

Functional studies of organisms and human models have revealed that epigenetic changes can significantly impact the process of aging. Non-coding RNA (ncRNA), one of epigenetic regulators, plays an important role in modifying the expression of mRNAs and their proteins. It can mediate the phenotype of cells. It has been reported that nc886 (=vtRNA2-1 or pre-miR-886), a long ncRNA, can suppress tumor formation and photo-damages of keratinocytes caused by UVB. The aim of this study was to determine the role of nc886 in replicative senescence of fibroblasts and determine whether substances capable of controlling nc886 expression could regulate cellular senescence. In replicative senescence fibroblasts, nc886 expression was decreased while methylated nc886 was increased. There were changes of senescence biomarkers including SA-β-gal activity and expression of p16INK4A and p21Waf1/Cip1 in senescent cells. These findings indicate that the decrease of nc886 associated with aging is related to cellular senescence of fibroblasts and that increasing nc886 expression has potential to suppress cellular senescence. AbsoluTea Concentrate 2.0 (ATC) increased nc886 expression and ameliorated cellular senescence of fibroblasts by inhibiting age-related biomarkers. These results indicate that nc886 has potential as a new target for anti-aging and that ATC can be a potent epigenetic anti-aging ingredient.

## 1. Introduction

Cellular senescence in an irreversible state following cell proliferation arrest has emerged as a potentially important contributor to tissue dysfunction and aging of organisms [1]. Senescence is characterized by high activity of senescence-associated beta-galactosidase (SA-β-gal), increase expression of senescence biomarkers such as p16INK4A and p21Waf1/Cip1, and decreased expression of LaminB1 [2]. There are two basic types of cellular senescence: replicative senescence and stress-induced premature senescence. Replicative senescence is defined as the phenomenon when normal cells stop dividing after reaching limited numbers of divisions. It is related to chronological aging. Continuous damage accumulation can induce replicative senescence of fibroblasts, leading to lost ability to remodel and organize the extracellular matrix (ECM). Main features of aged dermis are reduced amount of collagen fibers and increased production of matrix metalloproteinases (MMPs), which contribute to a thin and disorganized structure of dermis [3,4].

Epigenetic regulatory mechanisms are key mediators of the aging process, adapting to stressors and age-related changes in the genomic and molecular environment. These epigenetic alterations occur at various levels, including massive core histone reduction, deformation of histone by post-translational modification and DNA methylation, replacement of canonical histones with histone variants, and altered expression of noncoding RNA (ncRNA) during organismal and replicative senescence [5,6]. nc886 is 101 nucleotides long ncRNA that can bind to a target protein, thus mediating the activity of the protein and control gene expression [7]. nc886 has also been proposed as a tumor suppressor largely inferred by its expression pattern and its genomic location on human chromosome 5q31, the locus of the tumor suppressor gene. A feature of nc886 expression is the frequent silencing of malignancies by CpG DNA methylation in the promoter region [8]. Based on previous studies, we have found that the reduction of nc886 expression caused by UVB irradiation is associated with increases of COX-2 and MMP-9 through protein kinase RNA-activated (PKR) pathway in keratinocytes and that promotion of nc886 expression can be a useful strategy to develop UVB protective materials [9,10].

Green tea has been studied as a treatment for a variety of dermatological conditions, such as acne, rosacea, psoriasis, viral warts, and even skin cancer. Phenolic compounds including gallic acid (GC) and epigallocatechin gallate (EGCG) are well-known active compounds in green tea. (-)-Epigallocatechin-3-(3”-O-methyl) gallate (3”Me-EGCG) is a unique O-methylated form of EGCG and contained in oolong tea and green tea. In particular, Jangwon No. 3 (Amorepacific varieties of green tea) contains more 3”Me-EGCG than other varieties of green tea [11]. 3”Me-EGCG has been reported to exhibit antioxidant and photoprotective effects on keratinocytes [12]. In this study, we investigated the role of nc886 in replicative senescence of fibroblasts and determined whether highly concentrated green tea 3”Me-EGCG preparation (AbsoluTea Concentrate 2.0) that could increase nc886 expression could improve cellular senescence.

## 2. Results

### 2.1. Chemical Profile of AbsoluTea Concentrate 2.0 (ATC)

**Figure 1:**
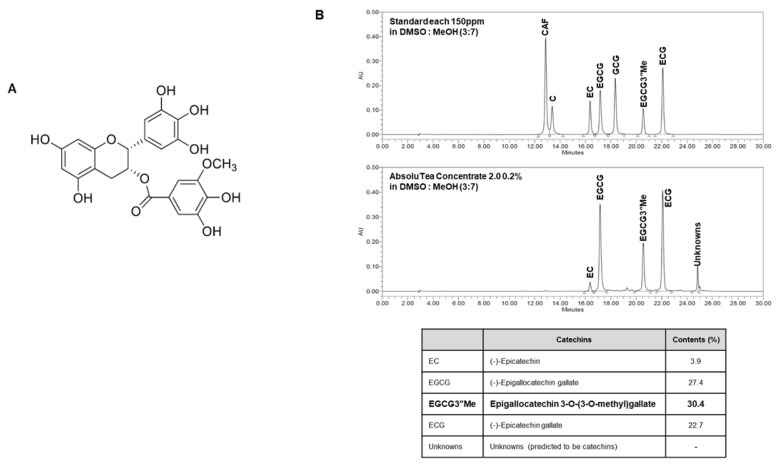
Structure of 3″Me-EGCG (**A**) and profile (**B**) of AbsoluTea Concentrate 2.0.

Chemical profile of AbsoluTea Concentrate 2.0 (ATC) is shown in Figure 1B. Results were obtained using HPLC for ATC prepared as described in the Method section. ATC contained 30% or more 3”Me-EGCG (Figure 1B). It is known that green tea extract does not contain 3”Me-EGCG or show a concentration of less than 6% [13,14]. Both young and senescent fibroblasts treated with ATC showed more increases of nc886 expression than those treated with 70% ethanolic extract of green tea (Supplementary Figure S1A). In addition, SA-β-gal expression levels in senescent fibroblasts treated with ATC were decreased more than in those treated with green tea extract (Supplementary Figure S1B).

### 2.2. nc886 Regulates Cellular Senescence of Fibroblasts

**Figure 2:**
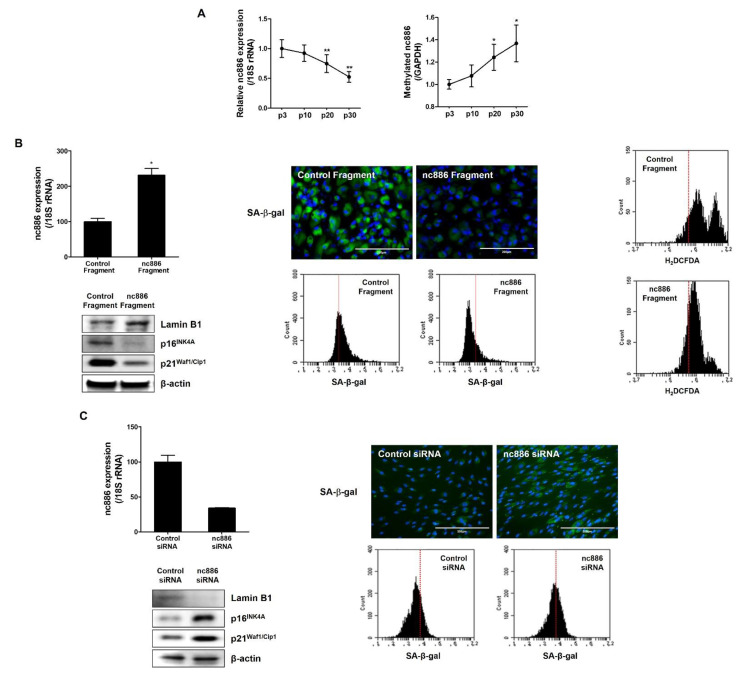
nc886 regulates cellular senescence of fibroblasts. (**A**) Expression of nc886 and methylated nc886 according to passage was confirmed by real-time PCR and normalized with 18s rRNA. (**B**) The nc886 fragment amplified and purified from human genomic DNA was transfected into senescent cells with Lipofectamine 3000. Overexpression was confirmed by observing the expression of nc886 by real-time PCR and SA-β-gal expression by fluorescence microscopy and flow cytometry. Expression of senescence marker protein and intracellular ROS generation were observed in nc886 overexpression model. (**C**) Cells were transfected with anti-oligos (si-ctrl and si-nc886) at a concentration of 250 ppm using Lipofectamin^TM^ RNAiMAX reagent. nc886 knock-down was confirmed by observing the expression of nc886 by real-time PCR and SA-β-gal expression by fluorescence microscopy and flow cytometry. The expression of senescence markers was confirmed with western blot. Scale bars: 200 μm. Data are presented as mean ± SD of three independent assays. *, *p* < 0.05; **, *p* < 0.01 compared to control.

To observe the role of nc886 in cellular senescence of fibroblasts, we used a replicative senescent model through growing and subculturing. As shown in Supplementary Figure S2, the activity of SA-β-gal and expression levels of senescent markers p16INK4A and p21Waf1/Cip1 were increased and Lamin B1 expression was decreased in senescent fibroblasts compared to young cells. Many previous studies have reported that aging is caused by oxidative stress. To confirm whether this series of oxidative stress is involved with cellular senescence, ROS levels in young (p3) and senescent fibroblasts (p30) were measured. Results showed that ROS levels were increased with aging (Supplementary Figure S2D). To observe potential impact of nc886 on cellular senescence in fibroblasts, nc886 gene expression level in each number of passages was determined. The level of nc886 expression was decreased with increasing passage number (Figure 2A). It has been reported that nc886 expression is decreased with the methylation of CpG islands. To determine whether nc886 downregulation in senescent fibroblasts was mediated by methylation of nc886, a methylation-specific PCR was performed. As shown in Figure 2A, methylation of nc886 was increased with increasing passage number (Figure 2A). This result demonstrates that the reduced expression of nc886 in a high passage number of fibroblasts is due to an increase of nc886 DNA methylation. To verify the role of nc886 in fibroblast senescence, changes of senescent markers in nc886 overexpression and knock-down models were determined. Results revealed that the group of senescent fibroblasts with nc886 overexpression showed decreased SA-beta-gal activity, decreased expression levels of p16INK4A and p21Waf1/Cip1, and increased level of laminB1. In addition, the increase of ROS in senescent fibroblasts was decreased by nc886 overexpression (Figure 2B). Conversely, in the group of young fibroblasts with nc886 knock-down, cellular senescence was accelerated and expression levels of cellular senescent markers such as p16INK4A and p21Waf1/Cip1 were increased (Figure 2C). These results suggest that nc886 can regulate cellular senescence of fibroblasts by regulating the expression of senescence markers (Figure 2B,C).

### 2.3. ATC Mitigates Cellular Senescence by Regulating nc886 Expression

**Figure 3:**
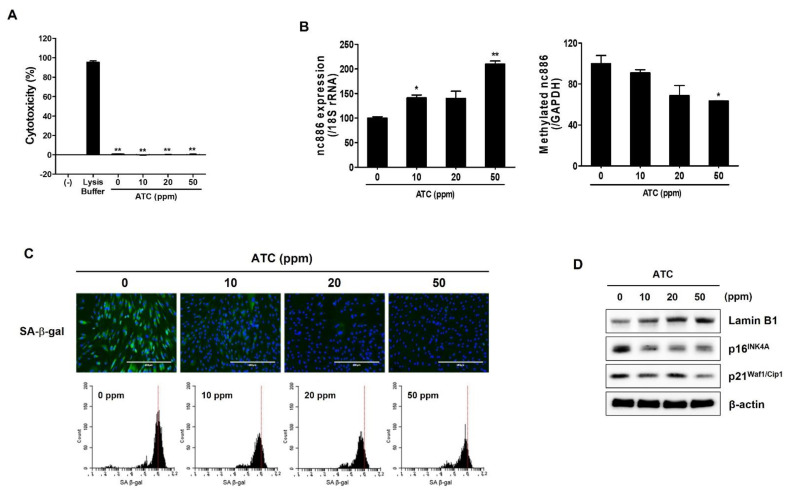
ATC increases nc886 expression in senescent fibroblasts. (**A**) Cytotoxicity was determined by LDH assay after treatment with ATC for 48 h. (**B**) Expression of nc886 was increased by treatment with ATC for 48 h in a concentration-dependent manner (0, 10, 20, 50 ppm). Cells were treated with previously described concentration of ATC and methylation of nc886 was confirmed. (**C**) Expression of SA-β-gal was confirmed in treated senescent fibroblasts via fluorescence microscopy and flow cytometry. (**D**) Expression of senescence markers. Scale bars: 200 μm. Data are presented as mean ± SD of three independent assays. *, *p* < 0.05; **, *p* < 0.01 compared to control.

Results obtained above showed that increasing nc886 expression could improve cellular senescence by mediating senescent markers. To observe the effect of ATC on nc886 expression, expression level and methylation of nc886 in senescent fibroblasts were evaluated after treatment with ATC at a non-cytotoxic concentration (Figure 3A). As shown in Figure 3B,C, ATC significantly increased nc886 expression and decreased methylation of nc886 in a concentration-dependent manner. This result demonstrated that increase of nc886 expression by ATC was mediated through inhibition of nc886 methylation. Additionally, we observed that ATC inhibited SA-β-gal activity and decreased expression levels of senescent markers p16INK4A and p21Waf1/Cip1 in senescent fibroblasts (Figure 3D,E). However, the expression of LaminB1 known to be decreased with aging, was increased in senescent fibroblasts treated with ATC (Figure 3E). These results demonstrate that ATC can mitigate cellular senescence by increasing nc886 expression.

### 2.4. ATC Regulates Age-Related Changes of ECM and SASP

**Figure 4:**
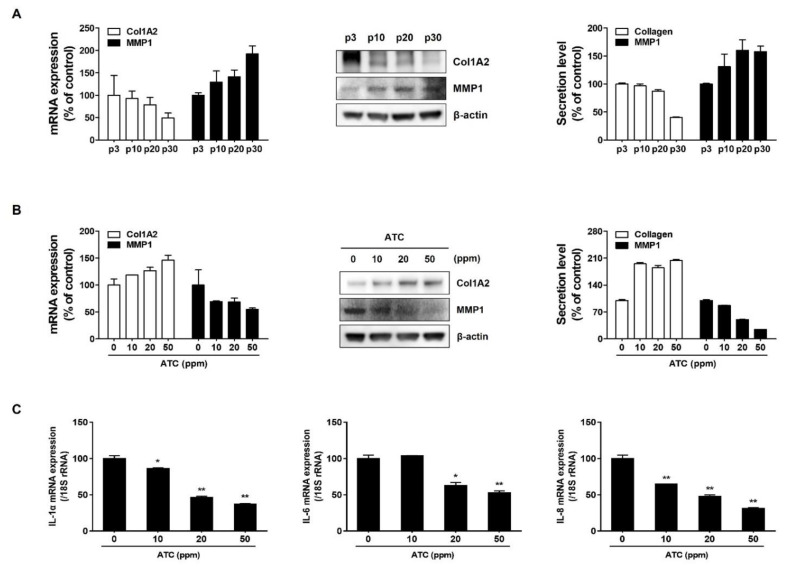
ATC regulates ECM related gene expression and SASP in senescent fibroblasts. (**A**) Representative expression of ECM related genes MMP1 and Collagen according to passage number was confirmed at RNA level, protein expression, and secretion. (**B**) Expression of ECM related genes MMP1 and Collagen after treatment with indicated concentration of ATC was confirmed at RNA level, protein expression, and secretion. (**C**) Representative expression of SASP related genes IL-1α, IL-6, and IL-8 according to passage number was confirmed at RNA level. Data are presented as mean ± SD of three independent assays. *, *p* < 0.05; **, *p* < 0.01 compared to control.

A characteristic of fibroblast senescence is that the activity of MMP-1, a component that degrades collagen, is increased, whereas collagen synthesis is decreased [15,16]. The increase of MMP-1 and the decrease of collagen were confirmed in senescent fibroblasts with high passage numbers (Figure 4A). However, ATC suppressed the expression of MMP-1 and increased collagen synthesis in senescent fibroblasts. This result indicates that ATC can improve age-related change of ECM through regulation of MMP-1 expression and collagen synthesis in senescent fibroblasts (Figure 4B). A senescence-associated secretory phenotype (SASP) in which senescent cells secrete specific pro-inflammatory factors, growth factors, and proteolytic enzymes has been reported to contribute to the onset and progression of aging-related diseases [17,18,19]. We observed the effect of ATC on SASP particularly pro-inflammatory cytokine such as IL-1α, IL_6, and IL-8. As shown in Figure 4C, ATC downregulated the expression levels of senescence-induced IL-1α, IL-6, and IL-8. This suggests that ATC can exerts an anti-aging effect through suppression of SASP.

## 3. Discussion

Senescent cells undergo characteristic morphological changes involving enlarged and often irregular nuclear and chromatin reorganization. When senescence is induced by DNA damage, replication depletion, or oncogene expression, Lamin B1 is primarily lost. Various internal or external stressors can trigger the DNA damage response (DDR) pathway to activate p53 and/or p16INK4A pathway. p16INK4A can inactivate Cdk4/6, result in the accumulation of phosphorylated pRb, disrupt regulation of E2F transcription factor, and induce cell cycle arrest or senescence [20,21]. Increased production of reactive oxygen species (ROS) mostly produced by dysfunctional mitochondria can lead to cellular senescence through DNA damage and transactivation of senescence signaling pathways, including p53, p16INK4A, and p21Waf1/Cip1 [22]. In this study, we found that nc886 expression was decreased with increasing passage number and that upregulation of nc886 expression mitigated cellular senescence via mediation of LaminB1, p16INK4A, p21Waf1/Cip1, and ROS production. nc886 is transcribed by RNA polymerase III (Pol III) and silenced by CpG DNA hypermethylation in many malignancies [8]. We observed that the expression of nc886 was decreased with methylation of CpG islands in senescent fibroblasts. This result indicates that hypermethylation of CpG islands impairs the functionality of nc886 as a suppressor of cellular senescence.

Specific gene expression pathways affected by nc886 are unclear. They are likely to include previously reported for other ncRNAs. nc886 can play critical roles in cellular senescence via directly or indirectly mediating the senescence signaling pathway at levels of chromatin structure, transcription factor activity, post-transcriptional, and post-translational gene regulation [23]. For example, nc886 can mediate the senescent pathway of cell cycle progression. It has been reported that AK156230 as a ncRNA is associated with the induction of replicative senescence of fibroblasts through its roles in autophagy and cell cycle progression. Downregulation of AK156230 induced cells to show senescence responses by activating p53 and p21 while decreasing cyclin-dependent kinase 1 (CDK1) [24]. Senescence-associated ncRNA1 (SAL-RNA1), MALAT1, and MIAT are known as negative regulators of cellular senescence. They are decreased in senescent fibroblasts. Downregulating of these genes can lead to increases of senescence markers such as SA-β gal, p16, p21, and p53 [25]. The effect of MALAT1 on cellular senescence was achieved through decline of oncogenic transcription factor b-Myb/Mybl2.

Although the expression of various ncRNAs is affected during senescence, only a few are functionally involved in senescence. Because localization of ncRNAs is important for understanding the mechanisms involved in function, nuclear and cytoplasmic ncRNAs are discussed separately when describing the molecular mechanisms affecting senescence [26]. The ncRNA located in the cytoplasm may function as a translation regulator through base pairing with the target mRNA [27]. ncRNA can affect protein expression levels by increasing or decreasing mRNA stability [28]. For example, during RAS-induced senescence, cytoplasmic UCA1 contributes to p16 mRNA stabilization by sequestering hnRNPA1 [29]. nc886 contains a protein kinase RNA-activated (PKR) binding site. PKR–nc886 binding can inhibit PKR activity. PKR, a cytoplasmic receptor for double-stranded RNA (dsRNA), is regulated by serine/threonine protein kinase that is activated by infection, cytokines, oxidative stress, and irradiation (including UV), leading to subsequent induction of inflammation and apoptosis [20,21] [30,31]. nc886 binds to PKR with an affinity comparable to dsRNA and prevents PKR from being activated. nc886 knockdown results in phosphorylation of eukaryotic initiation factor 2α subunit (eIF2α) via the PKR pathway, which causes cell death and inhibits global cellular protein synthesis. It has been reported that the production of COX-2, IL-8, and MMPs by TNF-α is mediated by the PKR pathway in human chondrocytes [32]. In a previous study, we have also demonstrated that UVB-induced inflammation can be regulated through the nc886-PKR pathway in keratinocytes [10]. Moreover, PKR is related to oxidative stress-induced injury in neonatal cardiac myocytes. Inhibition of PKR protects against H_2_O_2_-induced injury by attenuating apoptosis and inflammation [33]. Inflammation and oxidative stress are major factors that induce the aging process. Thus, the PKR pathway can mediate cellular senescence of fibroblasts induced by nc886 depletion. To elucidate the detailed regulatory mechanism involved in the effect of nc886 on cellular senescence of fibroblasts, further studies are needed.

Antiaging effects of green tea extract have been widely reported in previous studies. Antioxidant and anti-inflammatory activities of green tea are well-known mechanisms that contribute to its anti-aging effect. In a preliminary test, we found that ATC increased nc886 expression significantly more than 70% ethanolic extract of green tea (Supplement Figure S1A). This result suggests that concentrated catechins including 3”Me-EGCG can attribute to the upregulation of nc886 expression. To determine whether nc886 stimulating agent could prevent cellular senescence, we observed the effect of ATC on senescent fibroblasts. ATC increased the expression of nc886 by inhibiting the level of methylation of the nc886 gene in senescent fibroblasts (Figure 3B,C). ATC also relieved cellular senescence by mediating senescent biomarkers such as SA-β-gal activity, LaminB1, p16INK4A, and p21Waf1/Cip1 (Figure 3E). Moreover, increased MMP-1 and decreased collagen production induced by cellular senescence were restored by ATC (Figure 4B). SASP factors vary slightly depending on the type of cell and the type of aging-induced stress, they are commonly known as target genes for NF-κB that regulate pro-inflammatory cytokines such as IL-6 and IL-8. In this study, we confirmed that the increased expression of IL-1α, IL_6 and IL-8 in senescent cells was suppressed by ATC (Figure 4C). We cannot exclude the possibility that the improving effect of green tea on cellular senescence is achieved by multiple mechanism, including nc886 regulation, anti-oxidation, and anti-inflammation. However, our results suggest that increased nc886 by ATC plays a critical role in the suppression of cellular senescence, subsequently affecting ECM production of fibroblasts.

## 4. Materials and Methods

### 4.1. Preparation of ATC

Dried green tea leaves (*Camellia sinensis* var. sinensis cv. Jangwon No. 3) used for the current study were obtained from tea plants grown in Dolsongi tea garden (33°16′23.9″ N 126°28′58.3″ E), Jeju, South Korea. Dry tea leaves (170 g) were extracted with 70% EtOH (*v/v*) at room temperature for 16 h. The extract was then powderized using a lyophilizer (TF-10D, TEFIC BIOTECH, Xian, China). To prepare ATC, the EtOH extract was concentrated ten-fold with a rotary evaporator and loaded onto a column packed with AB-8 resin (inner diameter 5 cm, length 45 cm). Solvent elution of the column was performed with 4 times bed volume (BV) of 20%, 30%, and 100% EtOH (*v/v*). In this study, residual solvents in the column were removed with an air compressor before elution. The 30% EtOH eluate was concentrated twenty-fold and applied to a polyamide column (inner diameter 2.5 cm, length 40 cm). The polyamide column was eluted 5 times BV of 10% and 100% EtOH. The 100% EtOH eluate was carefully concentrated and lyophilized.

### 4.2. Cell Culture and Cell Treatment with ATC

Human dermal fibroblasts (HDFs) were purchased from ATCC (Manassas, VA, USA). Cell culture was conducted under controlled conditions at 37 °C with 5% CO_2_ using Dulbecco’s modified Eagle’s medium (WELGENE, Daegu, Korea) supplemented with 10% fetal bovine serum (Thermo Fisher Scientific, Waltham, MA, USA) and 1% of penicillin-streptomycin (WELGENE, Daegu, Korea). Cells were seeded into 6-well plates at density of 2 × 10^5^ cells/plate. After reaching 60% confluence, they were treated with indicated concentration of ATC for 72 h.

### 4.3. SA-β-Gal Staining and Flow Cytometry Analysis

Cells were stained using a SPiDER-β-Gal (Dojindo, Kumamoto, Japan) according to the manufacturer’s instructions. Cells were analyzed with an EVOS^®^ FL Cell Imaging System (Thermo Fisher Scientific, Waltham, MA, USA) and a BD FACS Calibur flow cytometer (BD Biosciences, Franklin Lakes, NJ, USA).

### 4.4. Intracellular ROS Measurement

The production of intracellular ROS was monitored using C2′,7′-dichlorodihydro- fluorescein diacetate (H_2_DCFDA; Molecular Probes), a fluorescent ROS indicator. Cells were incubated with H_2_DCFDA (5 μM) for 30 min at 37 °C. Cell-associated fluorescence was detected with a BD FACS Calibur flow cytometer (BD Biosciences, Franklin Lakes, NJ, USA).

### 4.5. Real-Time PCR and Methylation-Specific PCR

Total RNA was extracted using TRIzol (Thermo Fisher Scientific, Waltham, MA, USA). Quantitative measurement of RNA was performed using an Epoch microplate spectrophotometer (BioTek, Winooski, VT, USA). cDNA was synthesized using an am-fiRivert cDNA Synthesis Platinum Master Mix (GenDEPOT, Barker, TX, USA). Genomic DNA was extracted using the AccuPrep^®^ Genomic DNA Extraction Kit (BIONEER, Daejeon, Korea). Bisulfite-conversion was conducted using the EZ DNA methylation kit (Zymo Research, CA, USA). To perform qRT-PCR, an AMPIGENE^®^ cDNA Synthesis Kit (Enzo Life Sciences Inc., Farming-dale, NY, USA) and an ABI7500 real-time PCR system (Ambion Inc, Austin, TX, USA) were used. Primer sequences used in this study are described in Table 1.

### 4.6. Lactate Dehydrogenase Assay

LDH assay was used to determine the cytotoxicity by measuring lactate dehydrogenase (LDH) activity released from damaged cells. The assay was performed using an LDH Cytotoxicity WST Assay kit (Enzo life sciences, Farmingdale, NY, USA) according to the manufacturer’s instructions.

### 4.7. DNA Transfection for nc886 Overexpression and siRNA Transfection for nc886 Knockdown

To obtain DNA for overexpression of nc886 in cells, DNA was amplified with Accu-Power^®^ PCR PreMix (BIONEER, Daejeon, Korea) using genomic DNA of HDFs as template and the following primers described in Table 2.

Amplified DNA was purified using a QIAquick PCR Purification Kit (QIAGEN, Hilden, Germany). The purified DNA was transfected with a LipofectamineTM 3000 reagent (Thermo Fisher Scientific, Waltham, MA, USA). To knockdown nc886, cells were transfected with anti-oligos (si-ctrl and si-nc886) at a concentration of 250pM using the Lipofectamine™ RNAiMAX reagent (Thermo Fisher Scientific, Waltham, MA, USA).

### 4.8. Western Blotting

Cell lysates were prepared with PRO-PREP™ Protein Extraction Solution (iNtRON Bio-technology, Gyeonggi do, Korea). Total proteins were separated with a NuPAGE electrophoresis system (Thermo Fisher Scientific, Waltham, MA, USA) and transferred to polyvinylidene difluoride (PVDF) membranes. Immunoblotting was performed using primary antibody against p16INK4A, p21Waf1/Cip1 (Cell Signaling Technology, Danvers, MA, USA), MMP1, Col1A2, and β-actin (Santa Cruz Biotechnology, Inc., Dallas, TX, USA). Scanning densitometric values of bands were analyzed using the ImageJ, software version 1.52a (National Institutes of Health, Bethesda, MD, USA).

### 4.9. Enzyme-Linked Immunosorbent Assay

After indicated incubation, collagen (Procollagen Type I C-Peptide EIA Kit, Takara, Shiga, Japan) and MMP-1 (R&D Systems, Minneapolis, MN, USA) in culture supernatant were measured using the enzyme-linked immunosorbent assay (ELISA) kit following the manufacturer’s instructions.

### 4.10. Statistical Analysis

All results are presented as mean ± standard deviation (SD). Differences between two groups were evaluated by the *t*-test or analysis of variance (ANOVA) using GraphPad Prism. Statistical significance was indicated as either *p* < 0.05 or *p* < 0.01.

## 5. Conclusions

In conclusion, regulating nc886 expression can be a potential target of cellular senescence in fibroblasts and that ATC can be a potent epigenetic anti-aging ingredient.

## Figures and Tables

**Table 1:** Sequences of primers used Real-Time PCR and Methylation-Specific PCR.

Gene Name	Forward Primer	Reverse Primer
nc886	CGGGTCGGAGTTAGCTCAAGCGG	AAGGGTCAGTAAGCACCCGCG
p16INK4A	CTCGTGCTGATGCTACTGAGGA	GGTCGGCGCAGTTGGGCTCC
p21Waf/Cip1	CTCGTGCTGATGCTACTGAGGA	GGTCGGCGCAGTTGGGCTCC
Col1A1	GATTCCCTGGACCTAAAGGTGC	AGCCTCTCCATCTTTGCCAGCA
MMP1	ATGAAGCAGCCCAGATGTGGAG	TGGTCCACATCTGCTCTTGGCA
IL-1α	TGTATGTGACTGCCCAAGATGAAG	AGAGGAGGTTGGTCTCACTACC
IL-6	AGACAGCCACTCACCTCTTCAG	TTCTGCCAGTGCCTCTTTGCTG
IL-8	GAGAGTGATTGAGAGTGGACCAC	CACAACCCTCTGCACCCAGTTT
18S rRNA	CGGCTTTGGTGACTCTAGAT	GCGACTACCATCGAAAGTTG
Methyl-nc886	TTCGGGTCGGAGTTAGTTT AAGCG	AATAAACACC CGCGAATCTCG
GAPDH	CATCAAGAAGGTGGTGAAGCAGG	AGTGGTCGTTGAGGGCAATGC

**Table 2:** Sequences of primers used DNA Transfection for nc886.

Gene Name	Forward Primer	Reverse Primer
Control fragment	CAACCTTGCGTGGCGTGTGAACT	CACATTCACACCTGATTCTGG
nc886 fragment	CTGCTGGACCTAGGTAGACG	AATCCATAACGCACTCCGCG

## Data Availability

Data will be made available on request.

## Supplementary Materials

The following are available online at https://www.mdpi.com/article/10.3390/ijms222413673/s1.
