# Supplementary material for: nc886, a Non-Coding RNA, Is a New Biomarker and Epigenetic Mediator of Cellular Senescence in Fibroblasts
Source: Int J Mol Sci. 2021 Dec 20;22(24):13673. doi: 10.3390/ijms222413673 (PMC8705676; doi:10.3390/ijms222413673)
Supplement: Supplementary file 1 [file ijms-22-13673-s001.zip › ijms-1477369-supplementary.pdf]

Supplementary Data

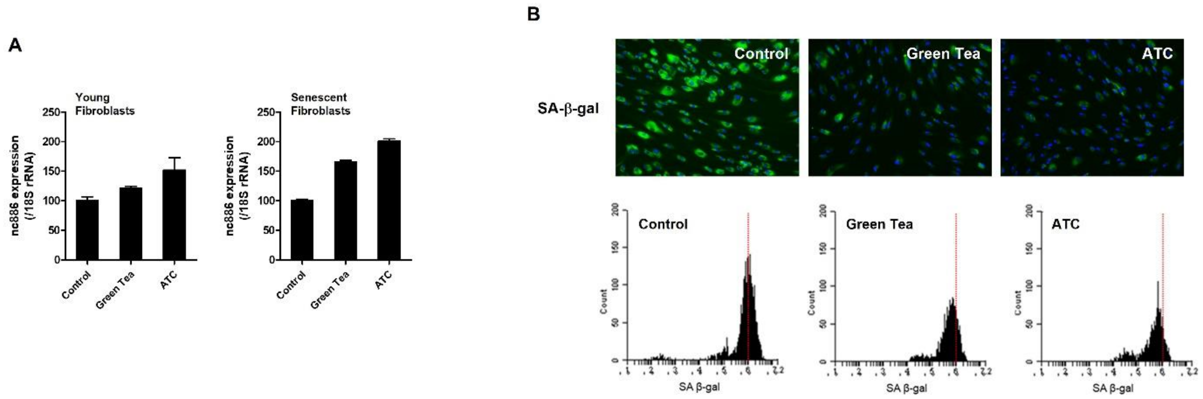

Figure S1. (A) Expression of nc886 according to passage (Young: p3, Senescent: p30) was confirmed by real-time PCR and normalized with 18s rRNA after treatment with green tea extract or ATC at 50 ppm for 48 hours. (B) Expression of SA-β-gal was confirmed in treated senescent fibroblasts via fluorescence microscopy and flow cytometry.

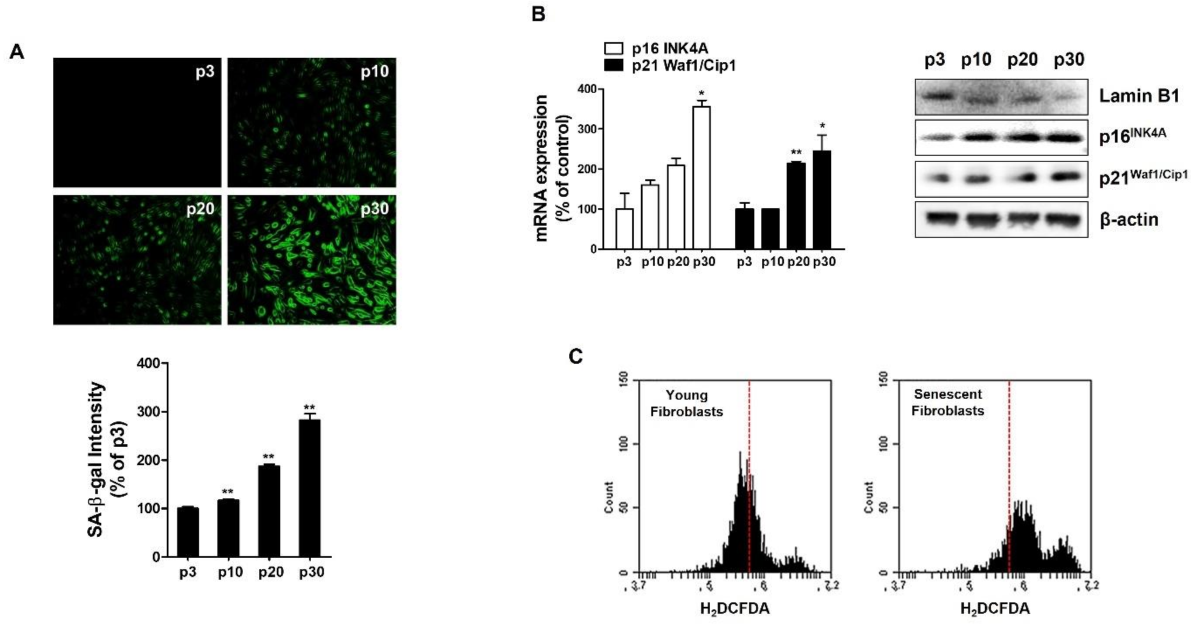

Figure S2. Changes of senescent biomarkers in replicative senescent model. (A) Expression of SA-β-gal in replicative senescent fibroblasts. Intensity of green fluorescence was presented in a bar graph. (B) Expression of Lamin B1, p16INK4A and p21Waf1/Cip1 as senescence markers at RNA and protein level. (C) Intracellular ROS generation in young (p3) and senescent (p30) fibroblasts were estimated by flow cytometry. Data are presented as the mean + SD of three independent assays. *p <0.05; **p <0.01 compared to control (p3).
